# The implicit and explicit attitudes of nursing students in Türkiye towards transgender individuals

**DOI:** 10.1192/j.eurpsy.2026.11948

**Published:** 2026-07-31

**Authors:** S. Karakaya Çataldaş, T. Şahin Tokatlıoğlu, A. Eminoğlu

**Affiliations:** 1 Faculty of Nursing, Koç University; 2 Faculty of Health Science/ Nursing, İstanbul Aydın University; 3 Faculty of Health Science/ Nursing, Marmara University, İstanbul, Türkiye

## Abstract

**Introduction:**

Transgender individuals face significant barriers in healthcare, including systemic discrimination and limited provider knowledge, which reduce access and quality of care. Nurses play a vital role in ensuring equitable treatment for all patients, yet their explicit (conscious) and implicit (unconscious) attitudes shape care quality. Although studies have examined nursing students’ attitudes toward sexual minorities, few explored implicit and explicit attitudes toward transgender individuals in Türkiye. Understanding these attitudes is crucial for addressing potential biases and promoting inclusive, transgender-affirming nursing education.

**Objectives:**

To examine the implicit and explicit attitudes of undergraduate nursing students in Türkiye toward transgender individuals and identify demographic and psychosocial factors associated with these attitudes.

**Methods:**

A descriptive cross-sectional design was employed with 344 nursing students from universities in İstanbul, Türkiye. Data were collected online via Qualtrics and Inquisit. Explicit attitudes were measured using the *Attitudes Toward Transgendered Individuals Scale* (ATTI; 20–100, higher = more positive), and implicit attitudes with the *Implicit Association Test* – transgender version (D-scores −2 to +2). Sociodemographic data included gender, age, birthplace, residence, religious commitment, and previous contact or training on transgender health. Analyses used t-tests, ANOVA, correlations, and stepwise regression. Ethical approval was obtained from Koç University (2024.417.IRB3.179).

**Results:**

Students showed moderately positive explicit attitudes (M = 60.48, SD = 15.53) but slightly negative implicit attitudes (M = 0.19, SD = 0.43). No significant correlation existed between explicit and implicit attitudes. Explicit attitudes were more positive among women, those born in Türkiye, residents of metropolitan areas, and students with transgender friends or training (p < .05). Religious commitment correlated negatively (r = −.37, p < .001). The regression model explained 30.6% of the variance (R² = 0.306). Implicit attitudes showed no demographic differences.

**Image 3:**
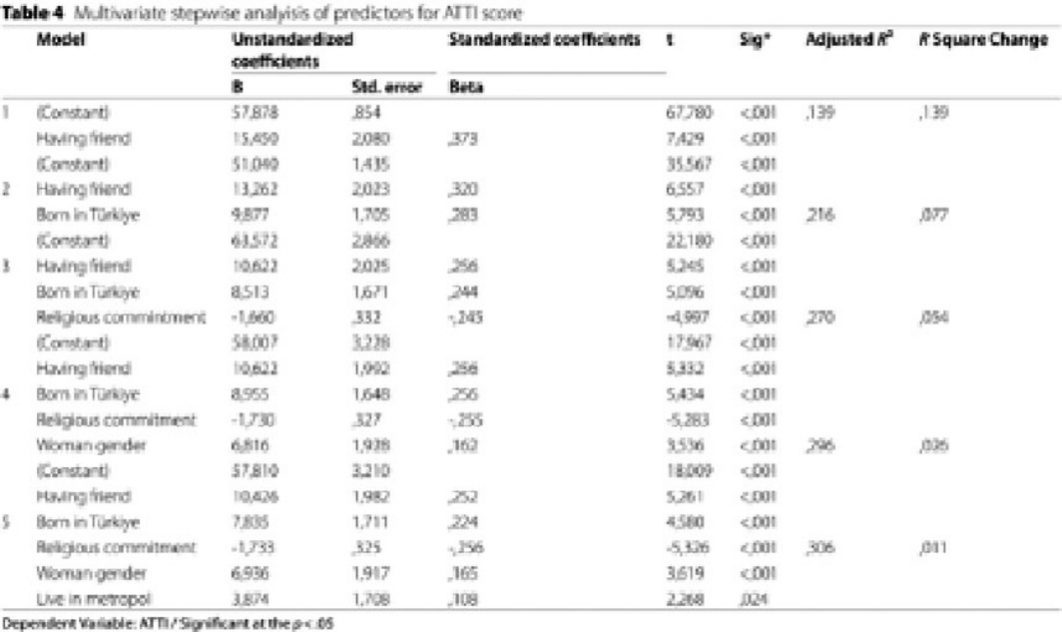
Image 3: Long description.

**Conclusions:**

Findings reveal a discrepancy between explicit and implicit attitudes: students consciously report acceptance yet retain subtle biases favoring cisgender individuals. Educational exposure and contact with transgender people predict positive attitudes. Integrating transgender-inclusive content and ethics-focused education in nursing curricula is essential to reduce bias, enhance equity, and improve healthcare experiences for transgender populations.

**Disclosure of Interest:**

None Declared

## Long descriptions

### Image 3: Long description

The table is titled Table 4 Multivariate stepwise analysis of predictors for A T T I score. It contains seven main columns: Model, Unstandardized coefficients (split into B and Std. error), Standardized coefficients (Beta), t, Sig asterisk, Adjusted R super 2, and R Square Change.

* Model 1: Includes (Constant) B 57.878 and Having friend B 15.450, Beta .373. Adjusted R super 2 is .139.

* Model 2: Adds Born in Türkiye B 9.877, Beta .283. Adjusted R super 2 increases to .216.

* Model 3: Adds Religious commitment B -1.860, Beta -.245. Adjusted R super 2 increases to .270.

* Model 4: Adds Woman gender B 6.816, Beta .162. Adjusted R super 2 increases to .296.

* Model 5: Adds Live in metropol B 3.874, Beta .108. Adjusted R super 2 reaches .308.

All predictors in all models show significance levels of p < .001, except for Live in metropol in Model 5 which is p = .024. The dependent variable is A T T I. Significance is noted at the p < .05 level.

Navigate back to Image 3:.

